# Epigenetic Reprogramming of Kaposi’s Sarcoma-Associated Herpesvirus during Hypoxic Reactivation

**DOI:** 10.3390/cancers14215396

**Published:** 2022-11-02

**Authors:** Rajnish Kumar Singh, Dipayan Bose, Erle S. Robertson

**Affiliations:** Department of Otorhinolaryngology-Head and Neck Surgery, Perelman School of Medicine, University of Pennsylvania, Philadelphia, PA 19104, USA

**Keywords:** KSHV, hypoxia, epigenetic reprogramming, reactivation

## Abstract

**Simple Summary:**

Epigenetic reprogramming of the KSHV genome plays a central role in lytic replication of Kaposi’s sarcoma-associated herpesvirus (KSHV). Hypoxia, a common phenotype of cancer cells, exerts a negative effect on DNA replication. Interestingly, KSHV is known to bypass this repression and undergo lytic replication. Therefore, we investigated epigenetic reprogramming of the KSHV genome under hypoxic conditions. The KSHV genome was enriched with both transcriptional activator and repressor modifications of histones due to the upregulated expression of the modifying enzymes in KSHV-positive cells grown in hypoxia. KSHV-encoded antigens were responsible for this increase in modified histone proteins. The differential enrichment of KSHV-encoded LANA and RTA in hypoxia were suggestive of their role in transcriptional regulation. Furthermore, analysis investigating enrichment of DNA polymerase 1α on the KSHV genome in conjunction with single molecule analysis of replicated DNA identified specific regions of the KSHV genome that may be critical for DNA replication in hypoxia.

**Abstract:**

The biphasic life cycle (latent and lytic) of Kaposi’s sarcoma-associated Herpesvirus (KSHV) is regulated by epigenetic modification of its genome and its associated histone proteins. The temporal events driving epigenetic reprogramming of the KSHV genome on initial infection to establish latency has been well studied, but the reversal of these epigenetic changes during lytic replication, especially under physiological conditions such as hypoxia, has not been explored. In this study, we investigated epigenetic reprogramming of the KSHV genome during hypoxic reactivation. Hypoxia induced extensive enrichment of both transcriptional activators and repressors on the KSHV genome through H3K4Me3, H3K9Me3, and H3K27Me3, as well as histone acetylation (H3Ac) modifications. In contrast to uniform quantitative enrichment with modified histones, a distinct pattern of RTA and LANA enrichment was observed on the KSHV genome. The enrichment of modified histone proteins was due to their overall higher expression levels, which was exclusively seen in KSHV-positive cells. Multiple KSHV-encoded factors such as LANA, RTA, and vGPCR are involved in the upregulation of these modified histones. Analysis of ChIP-sequencing for the initiator DNA polymerase (DNAPol1α) combined with single molecule analysis of replicated DNA (SMARD) demonstrated the involvement of specific KSHV genomic regions that initiate replication in hypoxia.

## 1. Introduction

Epigenetic reprogramming of Kaposi’s sarcoma-associated herpesvirus (KSHV) genome in infected cells is an essential event for achieving latency through control of viral gene expression [1,2]. In addition, reversing these epigenetic changes is anticipated to be a necessary event for controlling the process of latency and induction of lytic replication [2,3]. KSHV is the etiological agent of Kaposi’s sarcoma, primary effusion lymphoma, and multicentric Castleman disease [4,5], and exhibits the typical phases of latent and lytic cycle–like most herpesviruses [6,7]. Once inside a cell, the KSHV genome undergoes circularization and chromatinization before it attaches itself to the host genome to establish latency [7]. The major changes that occur during this event are the epigenetic reprogramming of the KSHV genome and its associated histone proteins [8]. The epigenetic reprogramming of the KSHV genome is central to the establishment and maintenance of latency after initial infection, which is important for controlling gene expression, and hence evading the host immune system [9,10]. The most established epigenetic modifications associated with the KSHV genome during the establishment of latency, or reversal from latent to lytic switch or vice versa, are the methylation of histones (H3K4Me3, H3K9Me3, H3K27Me3, H3K36Me3), histone acetylation (H3Ac), and DNA methylation [11,12]. Furthermore, the reversal of these acquired epigenetic modifications can lead to the transition from latency to lytic cycle replication [13]. The establishment of viral latency after initial infection, and reactivation from latency to lytic, is a natural phenomenon that follows a defined pathway [14]. In general, KSHV virions that infect a cell undergo latency. However, only a fraction of latently infected cells show characteristics of the lytic cycle during natural physiological conditions [15]. What exactly induces the latency of KSHV once it enters a cell is not fully understood, although several factors are known that can lead to the reactivation of KSHV from latency [16]. The physiological inducers of the KSHV lytic cycle are generally stress inducers such as radiation, metabolic stress, and hypoxia [2]. Direct inducers of the lytic cycle include chemical agents, which can directly influence epigenetic homeostasis of the genome, such as 12-O-Tetradecanoyl Acetate (TPA) and butyric acid [2,17,18]. Epigenetic changes that occur on the KSHV genome due to chemical induction, leading to the reactivation of the KSHV genome, are well-studied [19]. However, a complete understanding of the epigenetic reprogramming that occurs under physiological conditions such as that mediated by hypoxia, commonly referred to as hypoxic reactivation, has not been previously investigated [20,21]. Further, only few studies are available that investigate epigenetic changes on the KSHV genome during its reactivation from latency [3,22]. These studies are more focused on the analysis of the epigenetic landscape during either the establishment of latency after initial infection [23], or artificial conditions of chemically induced reactivation of KSHV [3,24]. Furthermore, the available studies are focused on investigating the correlation of transcriptional changes of KSHV during the establishment of latency or during chemical reactivation of KSHV. Hypoxic reactivation of KSHV has received more interest recently, as this represents a true physiological condition that occurs in naturally infected tumors in humans [20].

Hypoxic reactivation of KSHV itself represents an important phenomenon of the KSHV life cycle, where the viral genome undergoes productive replication after bypassing negative regulation of DNA replication [20,21]. It is important to note that hypoxia typically exerts repressive activities on cellular processes, which include replication [25,26], transcription, and translation [27,28,29], in addition to its detrimental role on ATP generation through oxidative phosphorylation [30]. Additionally, hypoxia induces large-scale degradation of cellular proteins involved in these processes [31,32]. Our previous studies have shown that KSHV reprograms cellular metabolism and modulates this cellular pathway to create replication-favorable conditions during its incidental encounter with the hypoxic microenvironment to drive productive replication [20,33,34]. In the present study, we investigated the reprogramming of the KSHV epigenome during hypoxic reactivation. In our initial experiments in KSHV-positive primary effusion lymphoma cell lines (BC3 cells), we observed an interesting phenomenon where the KSHV genome was enriched with different known modifications of histones such as H3K4Me3, H3K9Me3, H3K27Me3, and H3Ac. The multi-fold enrichment of these epigenetic modifications on the KSHV genome was a result of induced expression of these modifier proteins in hypoxia, exclusively in the background of KSHV-positive cells, where multiple KSHV-encoded antigens are expressed and contribute to the induction of epigenetic modifications. The observations were further confirmed by investigating levels of these modification in cell lines with isogenic background and similar passage numbers in the absence or presence of KSHV (BJAB and BJAB-KSHV cells, respectively) Moreover, differential enrichment of KSHV-encoded LANA or RTA at specific sites were more suggestive of transcriptional regulation. 

Direct ChIP- sequencing-based mapping with DNA polymerase (DNAPol1α), along with single molecule analysis of replicated DNA (SMARD), suggested an exclusive involvement of the KSHV genomic region at or near the terminal repeats. Other regions include the central genomic regions (co-ordinates 23466 to 23536, 58907 to 58979, 68333 to 68405, and 126233 to 126318) [20], as prominent origins of replication in hypoxia. Our data strongly suggest the differential epigenomic reprogramming of KSHV-positive cells under hypoxic conditions when compared to non-infected cells, and that KSHV-encoded factors play a critical role in modulating changes in the overall epigenome of infected cells in hypoxia. Furthermore, this work highlights specific origins of replication that can preferentially function in hypoxia. Finally, this study suggests that additional investigational efforts are needed to provide further insights into the role of KSHV and its encoded antigens on the replication of the host genome, as well as that of other viruses on epigenetic reprogramming of virus and host genomes in hypoxia.

## 2. Materials and Methods

### 2.1. Plasmid Constructs, Cell Culture, Transfection, RNA Isolation, cDNA Synthesis, and Real-Time PCR

BJAB, BJAB-KSHV, BC3, BCBL1, JSC1, Ramos, and DG75 cells were grown in RPMI medium containing 7% bovine growth serum (BGS) and Penicillin/Streptomycin at the recommended concentration, and maintained at 37 °C and 5% CO_2_. pBS-puroA (900 bp; terminal repeat sequence of KSHV), pBS-puroH (6 kb genomic fragment of KSHV; co-ordinate 26937–33194), pBS-puro-GA5 (10 kb genomic fragment of KSHV; co-ordinate 36883–47193), and Supercos1-GB22 (15 kb genomic fragment of KSHV; co-ordinate 85820–100784), were described earlier [20]. FLAG-tagged (pA3F-LANA), myc-tagged RTA (pEF-RTA), vGPCR (pCEFL-vGPCR), GFP-tagged vCyclin, and vFLIP (pLVX-ACGFP-vCyclin and pLVX-ACGFP-vFLIP, respectively) constructs were described earlier [20,21]. Transfection experiments were performed using jetPRIME reagent (Polyplus Transfection Inc., New York, NY, USA) according to the manufacturer’s protocol. RNA from cultures were isolated using Trizol reagent by the standard chloroform extraction method [34]. Next, 2 µg of RNA was used to synthesize cDNA using superscript cDNA synthesis kit (Invitrogen, Inc., Carlsbad, CA, USA). The synthesized cDNA was diluted 10 times with RNase free water, and 1 µL of the diluted cDNA (10 ng/µL) was used for subsequent real-time PCR. Sequences of real-time PCR primers with efficiency more than 90% are as follows: P4HA1 forward primer 5’-TGAGTTGGAGAATCTGGTCC-3’; P4HA1 reverse primer ‘5’–AGACGTAACAGAGCTTTGGC (amplicon size 123 bp); PDK1 forward primer 5’-GGATCAGAAACCGACACAAT-3’; PDK1 reverse primer 5’-ACATTCTGGCTGGTGACAGG (amplicon size 100 bp). The representative image for relative standard curve for the real-time PCR is provided in the Supplementary Figure S1. 

### 2.2. Western Blot

Whole cell lysates were prepared in RIPA buffer and separated on 10% polyacrylamide gel followed by transfer to a nitrocellulose membrane. Next, 5% skimmed milk was used for blocking at room temperature for 1 h with gentle shaking. Primary antibody against H3K4Me3, H3K9Me3, H3K27Me3, and H3Ac were procured from Millipore Inc. (Burlington, MA, USA). GAPDH and GFP antibodies were obtained from Santa Cruz Biotechnology Inc. (Dallas, TX, USA). Antibodies against DNMT1, DNMT3A, and DNMT3B were procured from Abcam Inc. (Cambridge, UK). The FLAG antibody was purchased from Sigma (Sigma Aldrich Inc., St. Louis, MO, USA). Antibodies against myc, LANA, RTA, and vGPCR were described earlier [20]. Nitrocellulose membranes with transferred proteins were incubated in primary antibody overnight at 4 °C with gentle shaking followed by washing with TBST three times (5 min each). Probing with IR conjugated secondary antibody was performed at room temperature for 1 h followed by washing three times (5 min each) with TBST. The membrane was scanned using an Odyssey scanner (LiCor Inc. Lincoln, NE, USA).

### 2.3. Pulse Field Gel Electrophoresis and Southern Blot

Cells were pulsed with 10 µM Chlorodeoxyuridine in cell culture medium for 4 h followed by pelleting down the cells at 1500 rpm for 5 min. Cells were further resuspended in fresh medium containing 10 µM Iododeoxyuridine and again pulsed for 4 h. At the end of pulsing, the cells were pelleted, washed with PBS, and finally resuspended in 0.5 mL PBS. Equal volumes of cell suspension and 1% Incert agarose were equilibrated at 45 °C for 5 min before mixing and molding in the cast for the preparation of cell embedded agarose plugs. The agarose plugs were digested in 0.5 M EDTA containing 1% Sarcosine and Proteinase K each for 96 h with a change of lysis buffer every 24 h. Proteinase K digestion was followed by washing in TE buffer pH8 for 72 h with a change of buffer after each 24 h. Final washing of plugs was in TE pH8 containing 1 mM PMSF. Pme1 was used to digest and linearize KSHV episomal DNA. Cell-embedded plugs were placed over comb fitted with agarose gel casting tray; 0.7% low melting agarose was poured, and the gel was left at 4 °C for polymerization. DNA was separated by Pulse field gel electrophoresis at 6 V/cm with an initial swing of 9 s and final swing of 14 s for 36 h on a CHEF-DRII electrophoresis system (Bio-Rad, Hercules, CA, USA). Post PFGE, Pme1 digested DNA in agarose gel was depurinated for 25 min by shaking in 0.25 M HCl at 70 rpm. The gel was rinsed twice with double distilled water followed by denaturation in 1.5 M NaCl/0.5 M NaOH for 25 min, twice. The gel was rinsed twice with double distilled water followed by neutralization in 1 M Tris/1.5 M NaCl for 25 min, twice. The gel was further rinsed with distilled water twice and equilibrated in 20× SSPE (175 g NaCl/27.6 g NaH_2_PO_4_/7.4G EDTA/8.5 g NaOH per liter) for 30 min before setting the alkaline transfer on the nylon membrane. Transferred DNA was subjected to UV cross-linking at 1400 joule with default settings. Prehybridization was performed at 42 °C in prehybridization buffer (6× SSPE, 10× Denhardt, 1% SDS, 200 µL denatured salmon sperm DNA). Hybridization probes were prepared by random priming, and hybridization was performed at 49 °C in pre-hybridization buffer devoid of salmon sperm DNA. The membranes were washed twice for 10 min each with low stringency buffer (6× SSPE/0.2% SDS) followed by 1 min high stringency buffer (1× SSPE/0.2% SDS). The membranes were wrapped in saran wrap and exposed to a sensitive plate followed by imaging using a Phosphorimager (GE Healthcare, Chicago, IL, USA).

### 2.4. Single Molecule Analysis of Replicated DNA

#### 2.4.1. KSHV Genome Spreading and Detection

The complete procedure for SMARD was described earlier [35]. Briefly, individual KSHV DNA molecules were visualized by spreading over silanized glass slides and probing with KSHV-specific probes. In brief, the region corresponding to the area of southern blot for KSHV DNA was excised from the gel and washed with TE buffer several times. The block was pre-incubated with digestion buffer (TE8/100 mM NaCl/0.1% β-Mercaptoethanol) for 2 h at 4 °C. Next, 100 µL of digestion buffer was added per 100 mg of equilibrated agarose block, and kept at 45 °C for 5 min prior to melting the agarose at 65 °C for 10 min. Agarose digestion was performed by adding 4 µL of Gelase enzyme (Epicenter Inc. Madison, WI, USA) and incubating at 45 °C overnight. YOYO-1 staining of DNA was performed for 1–2 h by adding 0.1 µL YOYO-1 and 5 µL of β-mercaptoehanol per 100 µL solution. YOYO-1-stained DNA was stretched over the slide by pipetting through one side of a cover slip placed over the slide. The stretching of the DNA was monitored by visualization using a fluorescent microscope. The area of the coverslip was marked with a diamond glass marker, and the coverslip was removed, followed by dipping the slides in methanol containing 0.1% β-mercaptoethanol for 10 min. The DNA on slides were denatured by dipping the slides in denaturing buffer (0.1 N NaOH/0.1% β-mercaptoethanol in 70% ethanol) for 12 min. DNA molecules were fixed for 5 min in fixation buffer (denaturing buffer containing 0.5% glutaraldehyde). Slides were washed serially in 70% ethanol (three times, 2 min each), 95% ethanol (once for 2 min) and 100% ethanol (once for 2 min). Biotinylated hybridization probes corresponding to KSHV genome (6 kb: 26937–33194; 10 kb: 36883–47193 and 15 kb: 85820–100784) were prepared by nick translation. Then, 50 ng biotinylated probes from each reaction were added to 100 µL hybridization buffer (40% formamide/1M NaCl, 1%SDS, 10% dextran sulfate, 5 mM tris pH7.4, 3 µL salmon sperm DNA) and floated into the chamber using cover slips covering the hybridization area. Hybridization was performed at 37 °C in a humidified chamber overnight. 

#### 2.4.2. Immunostaining and Fluorescence in Situ Hybridization

The cover slips used to create hybridization chambers were removed, and the slides were rinsed in 2× SSC/1% SDS. The slides were further washed for 5 min at 37 °C in 2× SSC/1% SDS. Non-specific hybridized probes were removed by washing the slides for 5 min in pre-equilibrated high stringency washing buffer (4× SSC/40%) at 45 °C. The slides were rinsed in 2× SSC/0.1% NP-40 at room temperature followed by washing in 4× SSC/0.1% NP-40 four times. Slides were then rinsed in 1× PBS/0.03% NP-40. The slides were dried, and blocking was performed by adding 20 µL blocking buffer (3% BSA/10% FBS in 1× PBS) for 30 min at room temperature in a humidified chamber. Cover slips were removed and 20 µL of detection mix 1 (1:15 dilution of Alexa 350 conjugated Neutr-avidin in blocking buffer) was added for each slide and covered with a cover slip. Cover slips were removed after 20 min by sliding through 1× PBS, 0.03% NP40 and rinsed in fresh 1× PBS, 0.03% NP40 followed by incubation with detection mix 2 (1:15 dilution of anti-avidin in blocking buffer) with a coverslip. After incubation, coverslips were again removed, followed by repeated incubation with detection mix 1. The coverslips were removed, followed by incubation with detection mix 3 (1:15 dilution of anti-avidin/1:7.5 dilution of mouse anti-IdU/ 1:7.5 dilution of rat anti-CldU in blocking buffer) at room temperature in humid chamber. The coverslips were again removed and rinsed in fresh 1× PBS, 0.03% NP40, followed by incubation with detection mix 4 (1:15 dilution of Alexa 350 conjugated Neutr-avidin/1:7.5 dilution of Rhodamine anti-mouse/1:7.5 dilution of Fluorescein anti-rat in blocking buffer) at room temperature in a humidified chamber with a coverslip. The coverslips were again removed by passing through 1× PBS, 0.03% NP40 and rinsed in fresh 1× PBS, 0.03% NP40. Mounting was completed by adding 15 µL Vectashield followed by covering with a 22 × 30 coverslip and sealing with colorless nail polish. Slides were kept at 4 °C for a couple of hours before capturing the images on a fluorescent microscope.

### 2.5. ChIP, ChIP-Sequencing, and ChIP-qPCR

Cells were grown in either normoxia or 1% O_2_ induced hypoxia. Cross-linking was performed by adding formaldehyde to a final concentration of 1% directly into the medium at room temperature. Cross-linking was stopped, and the cells were pelleted by centrifugation at 1500 rpm for 5 min and washed three times by resuspending and pelleting in 1X PBS. The cell pellets were resuspended in 1ml cell lysis buffer (5 mM PIPES pH8.0/85 mM KCl/0.5% NP-40) containing protease inhibitors (1 µg/mL Aprotinin/1 µg/mL Leupeptin/1 µg/mL Pepstatin and 1 mM PMSF). The cells were incubated on ice while homogenizing with several strokes by a Dounce homogenizer. Nuclei were pelleted by centrifugation at 5000 rpm and lysed by adding 1 mL of nuclear lysis buffer (50 mM Tris, pH8.0/10 mM EDTA/1% SDS containing the same protease inhibitors as that of cell lysis buffer. Nuclear lyses were performed for 10 min on ice. Chromatin were fragmented to an average size of 300–400 bp with a Branson sonifier with a microtip in 20 s bursts, followed by cooling on ice for a total sonication time of 3 min per sample. Debris was cleared by centrifugation at 15,000 rpm for 15 min at 4 °C. The supernatant was diluted five times with ChIP dilution buffer ([0.01% SDS/1.1% Triton X-100/1.2 mM EDTA, 16.7 mM Tris, pH 8.1/167 mM NaCl plus protease inhibitors), followed by clearing with 80 µL protein agarose A/G slurry. Antibody binding was performed overnight at 4 °C with gentle shaking, and the immune complexes were collected after 1 h of binding with 60 µL protein agarose A/G beads. The beads were washed, and elution was performed by adding 250 µL elution buffer (1% SDS/0.1 M NaHCO_3_). Formaldehyde crosslinking was reversed by adding 1 µL 10 mg/mL RNase and 5 M NaCl to a final concentration of 0.3 M, followed by incubating at 65 °C water bath for 4–5 h. The DNA was precipitated overnight, pelleted, and resuspended in 100 µL of nuclease free water. Next, 2 µL of 0.5 M EDTA, 4 µL 1 M Tris, pH6.5, and 1 µL of 20 mg/mL Proteinase K was added, followed by incubation for 1–2 h at 45 °C. The DNA was finally purified using a Qiagen column. Then, 2 µL of DNA was used in subsequent ChIP-qPCR. The sequence of the primers used for ChIP-qPCR are provided in Supplementary Table S1.

ChIP library preparation and adaptor ligation was performed using Illumina TruSeq ChIP sequencing sample preparation kit (Illumina, San Diego, CA) according to the manufacturer protocol. ChIP sequencing was performed at the University of Washington. The data were analyzed through CLC Bio software (Qiagen Inc., Hilden, Germany). In brief, the experiments were performed on BC3 cells grown under normoxic or hypoxic conditions. The antibodies used in these studies were against H3Ac, H3K4Me3, H3K4Me9, H3K4Me27, LANA, RTA, and DNA Pol1α. The experiments were performed in duplicate, and histograms for the representative experiments are shown. The ChIP-seq library was prepared using the Illumina ChIP sequencing sample preparation kit. The sample passed through initial quality check and were used for sequencing on the Illumina platform. The data were analyzed by commercially available software (CLC Bio, Qiagen Inc., Hilden, Germany). The ChIP-seq analysis workflow by CLC Bio runs on a default setting of the parameters and can be applied to any dataset. The spatio-temporal distribution of differential enrichment is used by the software to denote the regions with significant difference. The workflow includes five connected steps: (a) importing the raw data file in Fasta format; (b) mapping the read to the reference genome; (c) calling peaks; (d) visualizing results by creating a track list; and (e) extracting the sequence of the peak region. The ChIP sequencing libraries were prepared according to the manufacturer’s protocol. IgG control and input were also included in experiments as per the requirement of histone enrichment or transcription factor binding requirements. 

### 2.6. Statistical Analysis

All real-time PCR or Western blot experiments were performed at least in triplicate. ChIP-Seq analyses were performed according to the standard pipeline (CLC Bio, Qiagen Inc., Hilden, Germany). Significance was based on the fold change of the different sets of results and was calculated by using the student t-test. The differences were considered statistically significant when the *p*-value was <0.05.

## 3. Results

### 3.1. Hypoxia Induces Enrichment of Modified Histone Proteins on the KSHV Genome

**Figure 1:**
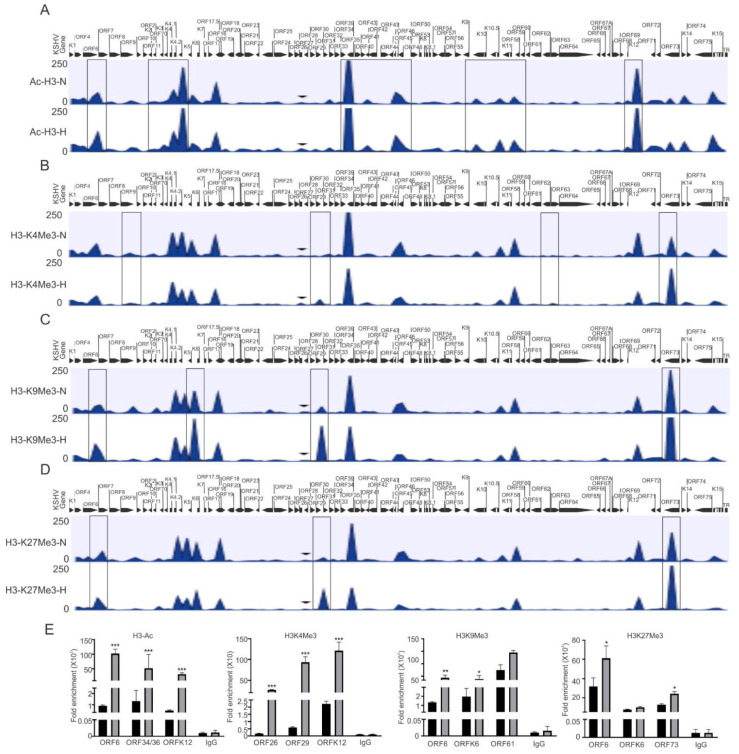
Differential enrichment of modified histone proteins on the KSHV genome during hypoxic reactivation. (**A**) The histogram for differential enrichment of AcH3 on the KSHV genome in normoxic and hypoxic conditions. (**B**) The histogram for differential enrichment of H3K4Me3 on the KSHV genome in normoxic and hypoxic conditions. (**C**) The histogram for differential enrichment of H3K9Me3 on the KSHV genome in normoxic and hypoxic conditions. (**D**) The histogram for differential enrichment of H3K27Me3 on the KSHV genome in normoxic and hypoxic conditions. A complete list of statistically significant enrichment region on the KSHV genome is provided as Supplementary File S1. (**E**) Validation of ChIP sequencing result using ChIP-qPCR. ChIP DNA was used to perform real-time PCR using primers specific to ORF6, ORFK6, ORF12, ORF26, ORF29, ORF34/36, ORF61, and ORF73 (N = Normoxia, H = Hypoxia). The *p* value of <0.05 was considered statistically significant. * *p* value < 0.05; ** *p* value < 0.01; *** *p* value < 0.005.

In the present study, we focused on investigating the epigenetic reprogramming of the KSHV genome under physiologically hypoxic conditions, with the aim of finding correlations between epigenetic changes of the KSHV genome and its replication strategy. We cultured KSHV-positive BC3 cells in either normoxic or hypoxic conditions, and cells were used to analyze the induction of hypoxia. As the Western blot analysis of HIF1α do not represent a suitable and reproducible marker for hypoxic induction in long-term hypoxic treatment experiment (Supplementary Figure S1A) [36], we confirmed hypoxic induction by analyzing the real-time expression of hypoxic markers that are represented downstream of HIF1α-activated genes including P4HA1 and PDK1 at the transcript level (Supplementary Figure S1B–E) [34,37]. After we confirmed the induction of hypoxia, the remaining cells were used for chromatin immunoprecipitation using antibodies against activating (acetylated H3 (H3Ac) and H3K4me3), and repressive (H3K9me3 and H3K27me3) histone modifications. The data were analyzed for differential enrichment on the KSHV genome using CLC Bio software, as described in the material and methods section. The status of histone modification enrichment on the KSHV genome in hypoxia was compared with cells cultured under normoxic conditions. The results of our analyses showed a phenomenon where several KSHV genomic regions were significantly enriched with these modifications, specifically under hypoxic conditions as compared to normoxic conditions (Figure 1A–D). Although this high enrichment was observed throughout the KSHV genome, the genomic region between ORFK1 to ORF10 (left end of KSHV genome) was observed to be dramatically enriched with these histone modifications as there were minimal modifications at this region of the KSHV genome in cells grown under normoxia (Figure 1A–D). A similar enrichment was observed between KSHV genomic region ORFK6 and ORF33, and between ORF61 and ORFK12 (Figure 1A–D). The regions with high enrichment due to these modifications are highlighted by boxes (Figure 1A–D). In fact, the genomic region corresponding to the latency locus were relatively less enriched with these modifications. Further, among the modifications examined, H3K27me3 and H3K9me3 modifications were even more highly enriched on the KSHV genome as compared to H3K4me3 or H3Ac (Supplementary File S1). A representative set of the ChIP-qPCR experiment was performed to validate the results obtained from ChIP sequencing. Briefly, primers against the KSHV genomic region with significant differential enrichment were used for subsequent qPCR using antibody-specific ChIP DNA. The ChIP-qPCR results confirmed the results obtained from ChIP sequencing experiments above (Figure 1E). Further, to rule out any technical artifacts, results taken after including the reading of IgG control, as well as data for some areas in ChIP-qPCR which either remained unchanged or reduced in occupancy, are provided (Figure 1E and Figure S1F).

### 3.2. KSHV Induces Expression of Modified Histone Proteins under Hypoxic Conditions

**Figure 2:**
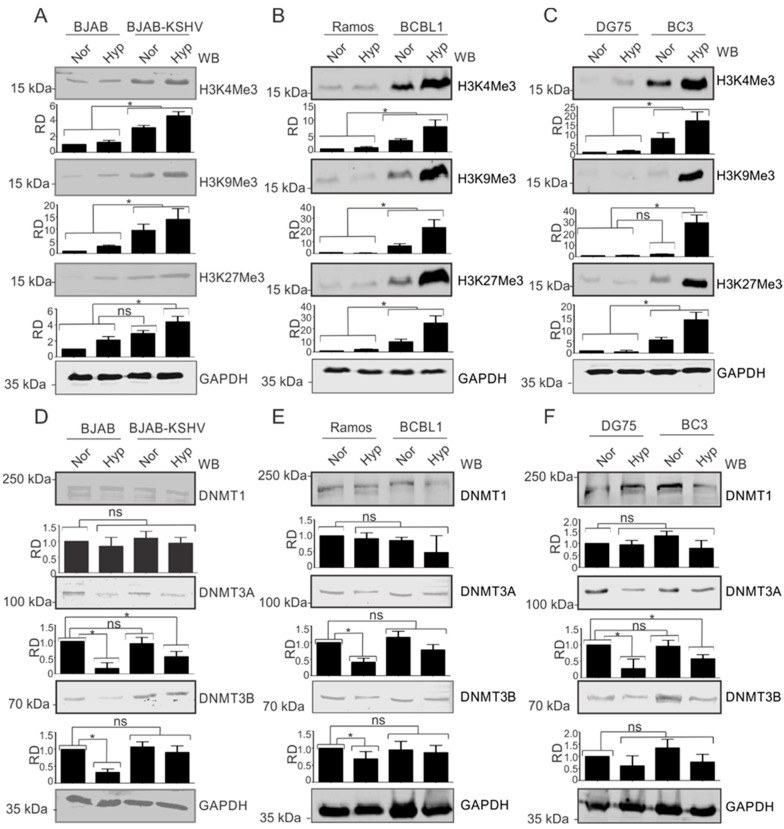
KSHV induces the expression of modified histone proteins. (**A**) Comparative analysis for levels of modified histone proteins in BJAB and BJAB-KSHV cells. Cells were grown either in normoxic or hypoxic conditions for 48 h. Equal amounts of proteins were used for analysis of the levels of H3K4Me3, H3K9Me3, and H3K27Me3 by Western Blotting. GAPDH served as loading control. (**B**) Comparative analysis for levels of modified histone proteins in KSHV-positive BCBL1 and KSHV-negative Ramos cells. Cells were grown either in normoxic or hypoxic conditions for 48 h. Equal amounts of proteins were used for analysis of the levels of H3K4Me3, H3K9Me3, and H3K27Me3 by Western Blotting. GAPDH served as the loading control. (**C**) Comparative analysis for levels of modified histone proteins in KSHV-positive BC3 and KSHV-negative DG75 cells. Cells were grown either in normoxic or hypoxic conditions for 48 h. Equal amounts of proteins were used for analysis of levels of H3K4Me3, H3K9Me3, and H3K27Me3 by Western Blotting. GAPDH served as a loading control. (**D**) Comparative analysis for levels of DNA modifying proteins in BJAB and BJAB-KSHV cells. Cells were grown either in normoxic or hypoxic condition for 48 h. Equal amounts of proteins were used for analysis of levels of DNMT1, DNMT3A, and DNMT3B by Western Blotting. GAPDH served as a loading control. (**E**) Comparative analysis for levels of modified histone proteins in KSHV-positive BCBL1 and KSHV-negative Ramos cells. Cells were grown either in normoxic or hypoxic conditions for 48 h. Equal amounts of proteins were used for analysis of levels of DNMT1, DNMT3A, and DNMT3B by Western Blotting. GAPDH served as loading control. (**F**) Comparative analysis for levels of modified histone proteins in KSHV-positive BC3 and KSHV-negative DG75 cells. Cells were grown either in normoxic or hypoxic conditions for 48 h. Equal amounts of proteins were used for analysis of levels of DNMT1, DNMT3A, and DNMT3B by Western Blotting. GAPDH served as a loading control (Nor = Normoxia, Hyp = Hypoxia). The *p* value of <0.05 was considered statistically significant. ns, not significant; * *p* value < 0.05.

The observation that modified histone proteins were significantly enriched on the KSHV genome in hypoxia allowed us to investigate the phenomenon in more detail. We asked where the observed modifications were occurring specifically on the KSHV genome or whether KSHV infection upregulated the basal expression levels of these proteins. For this, we used KSHV-negative BJAB and KSHV-positive BJAB-KSHV cells [33]. BJAB is a KSHV-negative Burkitt lymphoma cell line from which BJAB-KSHV cells were generated by stably transfecting BJAB cells with a recombinant KSHV bacmid clone [38]. The cells were grown in normoxia or 1% O_2_ to induce hypoxia, followed by investigation of the levels of modified histones such as H3K4Me3, H3K9Me3, H3K27Me3, and AcH3. The induction of hypoxia was confirmed by analyzing the real-time expression of P4HA1 and PDK1 (Supplementary Figure S2A,D). Interestingly, we observed that in KSHV-positive background (BJAB-KSHV cells), the levels of these modified histone proteins were dramatically upregulated by several fold (Figure 2A). To further corroborate the results observed in BJAB and BJAB-KSHV cells, we examined these changes in two different KSHV-positive pleural effusion lymphoma (PELs) cell lines–BCBL1 and BC3 [39]–and compared with KSHV-negative cell lines (Ramos and DG75) derived from patients with Burkitt’s lymphomas [20,21]. The induction of hypoxia in these cell lines was confirmed by analyzing the real-time expression of P4HA1 and PDK1 (Supplementary Figure S2B,C,E,F). As seen in the KSHV-positive BJAB cells with an isogenic background, the levels of modified histone proteins H3K4Me3, H3K9Me3, and H3K27Me3 were all strongly upregulated in KSHV-positive PEL cell lines (Figure 2B,C). This strongly demonstrated that KSHV contributes to the induction of modified histones in the hypoxic microenvironment. 

### 3.3. KSHV Modulates DNA Methyl Transferases in Hypoxia

We also wanted to know if these changes were just histone modification, or if methylation of the DNA was also affected as a result of changes in the modifying enzymes responsible for methylation of the viral and host genomic DNA (Figure 2D–F). Therefore, we included three well-known DNMTs in our western blot analyses. Notably, the levels of DNMTs showed negligible change between KSHV-positive or -negative cells, which was also true under conditions of hypoxia when compared to normoxia, especially for DNMT1. As BJAB-KSHV cells are not naturally infected cells, but rather generated by transfection of the KSHV genome in BJAB cells, we wanted to also corroborate these observations in naturally infected KSHV-positive PEL cells. The results showed that DNMT3A and DNMT3B levels were marginally stabilized in hypoxia in BJAB-KSHV cells compared to BJAB cells without KSHV where the levels were significantly reduced (Figure 2D). The results in naturally infected KSHV-positive PEL cell lines BC3 and BCBL1 grown under normoxic conditions were either marginally elevated or did not show any significant differences when compared with two other KSHV negative B cell lines, Ramos and DG75. When comparing the levels of these DNA methylases in BJAB and BJAB-KSHV cells, BCBL1 and Ramos or BC3 and DG75, stabilized expression of DNMT3A or DNMT3B was observed specifically in KSHV-positive background in hypoxia (Figure 2D,F). However, the levels of these proteins were either significantly reduced or unchanged in the KSHV-negative background under hypoxic conditions. This suggests that KSHV itself may drive the induced levels of DNMT3A and DNMT3B in normoxia, but in PEL cells there may be other compensatory activities that ultimately modulate the expression of these proteins and their impact on the KSHV genome during hypoxic reactivation.

### 3.4. KSHV-Encoded Antigens Modulates Expression of Histone Proteins and DNA Methylases

**Figure 3:**
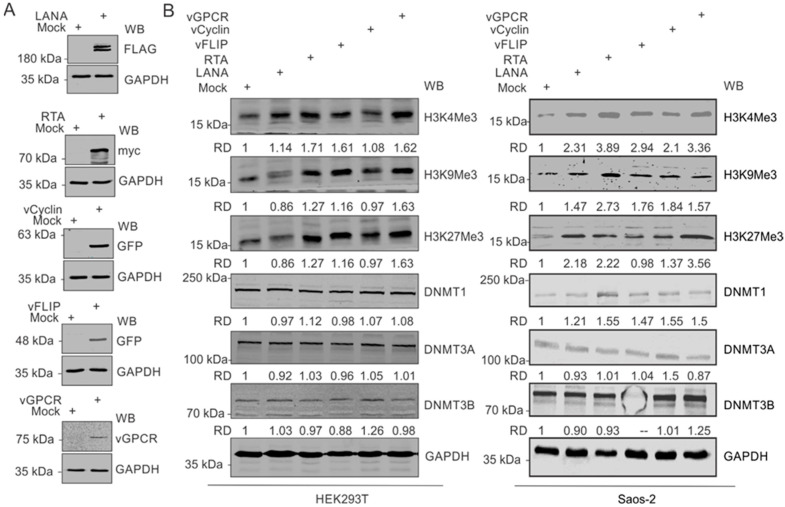
KSHV-encoded genes mediate the upregulation of modified histone proteins. (**A**) Representative image for the confirmation of ectopic expression of KSHV-encoded genes in HEK293T cells. (**B**) (Left panel): Western blot analysis for the expression of HEK4Me3, H4K9Me3 H3K27Me3, DNMT1, DNMT3A, and DNMT3B in HEK293 cells expressing KSHV-encoded LANA, RTA, vCyclin, or vGPCR proteins: 48 h post-transfection, cells were lysed, and equal amounts of whole-cell lysate were electrophoresed on polyacrylamide gels followed by transfer to nitrocellulose membrane. Expression levels of HEK4Me3, H4K9Me3, and H3K27Me3 were monitored by Western blot analysis using specific antibodies against respective proteins. GAPDH served as a loading control. (Right panel): Western blot analysis for the expression of HEK4Me3, H4K9Me3, and H3K27Me3, DNMT1, DNMT3A, and DNMT3B in Saos-2 cells expressing KSHV-encoded LANA, RTA, vCyclin or, vGPCR protein levels: 48 h post-transfection, cells were lysed, and equal amounts of whole-cell lysates were electrophoresed on polyacrylamide gel followed by transfer to nitrocellulose membrane. Expression levels of HEK4Me3, H4K9Me3, and H3K27Me3, DNMT1, DNMT3A, and DNMT3B were monitored by Western blot analysis using specific antibodies against respective proteins. GAPDH served as a loading control.

Based on our observation that upregulated expressions of modified histone proteins are predominantly in the KSHV-positive background, we hypothesized that one or more KSHV-encoded antigen may be a critical player in this phenomenon. Furthermore, since hypoxia remains a critical factor in the upregulation of these modified histone proteins, we focused on genes known to have differential expression in hypoxic conditions. These well-known KSHV-encoded genes with differential expression in hypoxic conditions include LANA, vCyclin, vGPCR, and vFLIP [33,40]. We also included KSHV-encoded RTA based on its critical role in KSHV reactivation [2]. Expression of these KSHV-encoded genes in HEK293T cells in culture was confirmed by Western blot against the epitope-tagged fusion of these proteins (Figure 3A). HEK293T cells were used in the experiment since it allowed for a reasonably high transfection efficiency of transfection and expression of cloned genes. A comparative analysis to determine the expression levels of modified histone proteins between mock transfected and KSHV-encoded protein-expressing cells suggested that multiple KSHV-encoded antigens were involved in the upregulation of the modified histone proteins (Figure 3B, Left panel). Expression of H3K4Me3 was minimally affected by the expression of LANA and vCyclin but had a more pronounced effect with the expression of RTA, vFLIP, and vGPCR. Expression of H3K9Me3 and H3K27Me3 was preferentially affected by RTA and vGPCR. However, the expression of DNMTs were mostly unchanged by the expression of KSHV-encoded proteins (Figure 3B, left panel). We further examined these results by expressing the same KSHV antigens in Saos-2 cells, an osteosarcoma cell line, which also represents a cell culture model for high efficiency transfection experiments. A similar pattern was seen in Saos-2 as well as with RTA, vFLIP and vGPCR showing a more prominent induction of histone modifications at H3K4, H3K9, and H3K27 for methylation at the 3 position (Figure 3B, right panel). RTA also showed a slightly elevated DNMT1, and vCyclin and vGPCR induced a moderate increase in DNMT3B. Little or no change was seen for DNMT3A across any of the KSHV antigens expressed (Figure 3B, right panel). 

### 3.5. Preferential Utilization of KSHV Replication Origins during Hypoxic Reactivation

**Figure 4:**
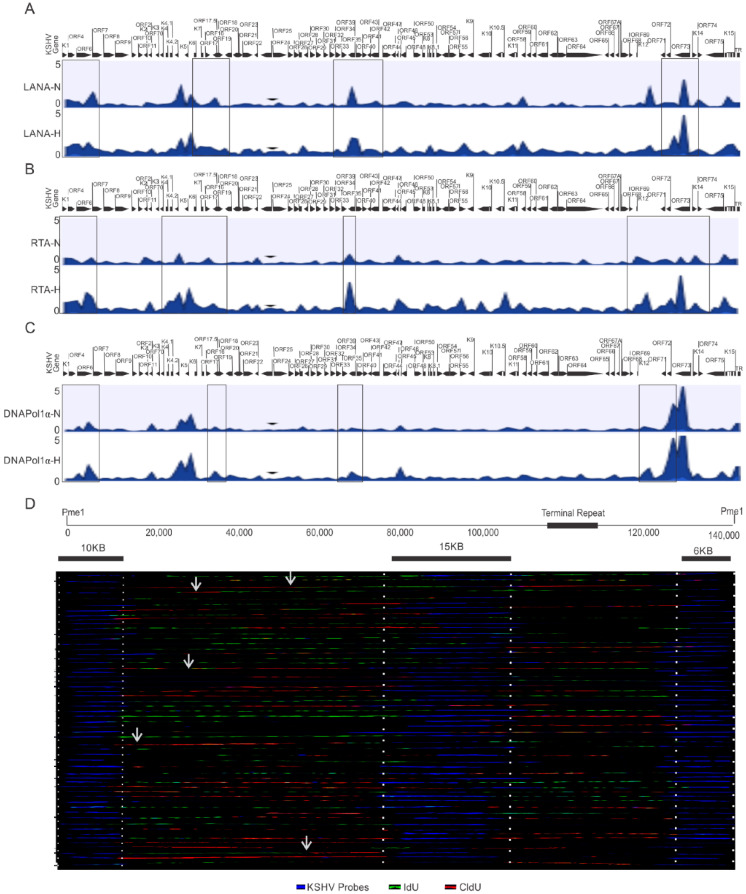
Identification of preferential regions of the KSHV genome for initiation of replication under hypoxic conditions. (**A**–**C**) Differential enrichment of LANA, RTA, and DNA Polymerase 1α on the KSHV genome in normoxic and hypoxic conditions. A complete list of statistically significant enrichment regions on the KSHV genome is provided as Supplementary File S1. (**D**) Single Molecule analysis of replicated DNA for identification of regions of nucleotide incorporation within the KSHV genome. Representative images for single molecule analysis of replicated DNA are shown for cells grown under hypoxic conditions. Blue fluorescence represents KSHV-specific probes; red represents the incorporation of IdU, while green represents CldU incorporation. Arrows represents areas of incorporation of the second pulsed nucleotide analog (N = Normoxia, H = Hypoxia).

One critical question that arises from these studies is whether KSHV utilizes different origins of replication during its reactivation under hypoxic condition. The ChIP-sequencing results using HEK4Me3, H4K9Me3, AcH3, and H3K27Me3 or DNMTs did not provide conclusive answers as to the identification of any specific origin of replication activated during hypoxic reactivation of KSHV. Therefore, we extended these studies using LANA, RTA and DNAPoL1α to determine whether there were genomic regions that preferentially recruited these proteins for replication at a specific site during hypoxia. KSHV-encoded LANA is involved in the recruitment of DNA polymerase clamp loader for efficient replication of KSHV [6,41]. We therefore decided to perform a ChIP-sequencing experiment with LANA to identify preferential genomic regions for clamp loading. Additionally, RTA was included in the ChIP-sequencing experiment based on its indispensable role in KSHV reactivation [2]. Furthermore, we included DNA polymerase 1α, a rate-limiting DNA polymerase due to its pivotal role in the initiation of DNA replication of the viral genome [41,42]. Analysis of the preferential enrichment of LANA on the KSHV genome indicated its relatively high enrichment near the terminal regions of the viral genome and throughout the central region (between ORF40 and ORF K10) (Figure 4A, top panels). A similar enrichment of RTA was observed on the KSHV genome near the terminal regions (Figure 4A, middle panel), but as compared to LANA, RTA enrichment on the KSHV genome was more concentrated on the left to central genomic region (between ORFK5 and ORF27). Interestingly, high RTA enrichment was also observed within the latency locus in hypoxia when compared to normoxic conditions, which showed LANA enrichment. Moreover, LANA enrichment was diminished at the latency locus in the cells grown under hypoxic conditions compared to normoxic conditions. Nevertheless, analysis of LANA and RTA enrichment on the KSHV genome were more suggestive of the involvement of larger genomic regions rather than concise genomic regions or sequences that could function as the origins of replication under hypoxic conditions. 

Finally, we looked on the differential enrichment of DNA polymerase 1α, in conjunction with that seen for LANA and RTA enrichment on the KSHV genome. The results of the DNA polymerase 1α clearly identified a more limited association in its interaction with the KSHV genomic DNA under hypoxic conditions. Our ChIP-sequencing analysis with DNA polymerase 1α identified 16 regions on the KSHV genome in hypoxia (Table 1). However, our ChIP-sequencing analyses for DNA polymerase 1α identified 27 regions on the KSHV genome in normoxia. (Table 2). These findings are congruent with previous studies, which demonstrated that KSHV can utilize multiple origins of replication during latent replication in normoxia [35]. Furthermore, when the level of DNA polymerase 1α enrichment at these regions were calculated to determine the significance based on the *p*-value (<0.05), four regions of the KSHV genome were identified that were more precise. These regions included coordinates at or adjacent to the terminal repeats 953 to 1028, between coordinates 23466 to 23536, between coordinates 58907 to 58979, and between coordinates 126233 to 126318 (Table 1).

To determine if these genomic regions of KSHV were the possible origin of replication, we performed a single molecule analysis of replicated DNA (SMARD) to directly visualize the incorporation of nucleotide analogs at regions that are activated origins of replication (Figure 4B). As compared to multiple known genomic regions of nucleotide incorporation on the KSHV genome in normoxic conditions [35], in hypoxia, only a limited number of regions were preferentially identified as active sites of incorporation for the labeled nucleotides (Figure 4B). This indicates that there are more focused regions of active incorporation of nucleotides on the KSHV genome during hypoxia, and that KSHV may preferentially utilize these regions during hypoxia to bypass checks to replication of its genome.

## 4. Discussion

Hypoxia is a detrimental stress for eukaryotic cells and imposes a great physiological challenge to DNA replication [25,43,44]. KSHV, an oncogenic herpesvirus, can bypass the challenges of hypoxia-induced repression of DNA replication, and proceed to productive replication [45,46]. In the past, we substantially described the mechanism of bypassing hypoxia-induced repression of DNA replication by KSHV [20,21]. Epigenetic reprogramming of the KSHV genome is an essential event to establish latency after initial infection of cells or to revert from latency to lytic phase [23,47]. In the present study, we wanted to investigate the unexplored question of epigenetic reprogramming of the KSHV genome during reactivation under the physiological condition of hypoxia. The results of our initial experiment with KSHV-positive BC3 cells, which showed a dramatic enrichment of histone modification observed during KSHV reactivation (H3Ac, H3K4Me3, H3K9Me3, and H3K27Me3), encouraged us to investigate this question in greater detail. The phenomenon of unexpectedly high enrichment of histone modification on the KSHV genome of BC3 cells under hypoxic conditions also allowed us to question whether the phenomenon is exclusive to KSHV-positive cells or if it happens to all types of cells grown under hypoxic conditions. To answer this, we used isogenic BJAB and BAB-KSHV cells with a similar passage number. Only BJAB-KSHV cells grown under hypoxic conditions showed a highly induced expression of modified histone proteins. This was further confirmed by comparing KSHV-positive PEL cells with other KSHV-negative B-cells grown under normoxic or hypoxic conditions. Interestingly, levels of these histone-modified proteins were lower in KSHV-negative cells when grown in hypoxia as compared to their normoxic counterparts. This suggested that KSHV-encoded factors may be involved in driving the upregulation of modified histone proteins. Subsequently, our results confirmed the involvement of multiple KSHV-encoded factors such as LANA, vGPCR, and RTA. However, the expression of these modified histone proteins was either unaffected or negatively affected in cells expressing KSHV-encoded vCyclin or vFLIP. KSHV-encoded LANA, RTA or vGPCR works positively to upregulate hypoxia inducible factor 1 alpha (HIF1α), either at transcription or post transcriptional levels [40,48], while vCyclin can negatively regulate HIF1α by mediating its lysosomal degradation in hypoxic conditions [20]. This allowed us to develop a hypothesis whereby HIF1α plays a definite role in this phenomenon.

KSHV utilizes multiple origins of replication under hypoxic condition for its latent replication [35], while during lytic replication it preferentially utilizes genomic regions located between K4.2 and K5, and between K12 and ORF71 [49,50]. Nevertheless, in our attempt to analyze the differential enrichment of modified histone proteins on the KSHV genome grown under hypoxic conditions, no specific origin of replication was identified to be activated during hypoxic reactivation of KSHV. Moreover, the analysis was more suggestive of a role for differential enrichment of modified histone proteins on transcriptional regulation. An experimental setup for concomitant analysis of the transcriptional profile of KSHV-encoded genes with this differential enrichment would provide better clarity of the interdependent events of epigenetic and transcriptional reprogramming of the KSHV genome under hypoxic conditions. Nevertheless, we further extended the study to map out specific origins of replication activated during hypoxic reactivation of KSHV. We performed the ChIP-sequencing experiment with DNA polymerase involved in the initiation of DNA replication (DNA polymerase 1α) along with single molecule analysis of DNA replication (SMARD). It is important to note that in non-infected cells, DNA polymerase 1α becomes degraded under hypoxic conditions, while KSHV-encoded LANA protects it from hypoxia-mediated degradation [21]. This phenomenon represents another example of the detrimental effect of hypoxia on DNA replication and how KSHV can bypass hypoxia induced repression of DNA replication. The combined analysis of DNA polymerase 1α ChIP-sequencing and SMARD results suggested preferential involvement of two KSHV genomic regions at or near the terminal repeats (between 953 and 1028), and near central region (between 68333 and 68405) as regions with potential for the initiation of DNA replication under hypoxic conditions. The combined findings of this present work with our previous reports–which included metabolic reprogramming of host cells by KSHV in hypoxic conditions, protection of cellular replication associated proteins from hypoxia mediated degradation, or the rescue of hypoxia-mediated repression of DNA replication by cycling HIF1α protein–provide a much broader vision, as well as greater insights into the events that occurs during hypoxic reactivation of KSHV.

## Figures and Tables

**Table 1:** Enrichment of DNA Polymerase 1α on KSHV genome in hypoxia.

DNA Pol 1A Hypoxia								
Region	Center of Peak	Length	Peak Shape Score	*p*-Value	5′ Gene	5′ Distance	3′ Gene	3′ Distance
953..1028	994	76	1.61	0.05	K1	0	ORF4	23
4689..4752	4721	64	1.54	0.06	ORF6	0	ORF7	1841
5314..5381	5345	68	1.69	0.05	ORF6	0	ORF7	1212
21,219..21,293	21,259	75	1.36	0.09	ORF70	182	K4	0
22,634..22,704	22,669	71	1.54	0.06	K4.1	172	K4.2	0
23,466..23,536	23,501	71	2.74	3.09 × 10^−3^	K4.2	387	K5	2328
25,739..25,802	25,769	64	1.38	0.08	K4.2	2660	K5	62
30,816..30,888	30,850	73	1.56	0.06	ORF16	0	ORF17.5	0
58,907..58,979	58,942	73	3.33	4.35 × 10^−4^	ORF38	0	ORF39	20
68,333..68,405	68,367	73	1.91	0.03	ORF44	933	ORF45	0
85,615..85,692	85,650	78	1.53	0.06	vIRF-1	199	vIRF-4	413
90,920..90,989	90,951	70	1.67	0.05	vIRF-4	1747	vIRF-3	0
119,641..119,713	119,675	73	2.37	8.83 × 10^−3^	HHV8_gs02	33	HHV8_gs03	520
124,840..124,931	124,897	92	1.53	0.06	HHV8GK18_gp82	1024	HHV8GK18_gp81	0
126,233..126,318	126,284	86	4.39	5.71 × 10^−6^	HHV8GK18_gp82	2417	HHV8GK18_gp81	0
135,235..135,325	135,291	91	1.34	0.09	K15	0		

**Table 2:** Enrichment of DNA Polymerase 1α on KSHV genome in normoxia.

DNA Pol 1A Normoxia								
Region	Center of Peak	Length	Peak Shape Score	*p*-Value	5′ Gene	5′ Distance	3′ Gene	3′ Distance
2434..2511	2461	78	1.69	0.05	ORF4	0	ORF6	667
4688..4761	4712	74	1.69	0.05	ORF6	0	ORF7	1832
5308..5387	5344	80	2.59	4.78 × 10^−3^	ORF6	0	ORF7	1206
21,214..21,311	21,261	98	1.39	0.08	ORF70	177	K4	0
22,640..22,711	22,665	72	2.17	0.01	K4.1	178	K4.2	0
23,467..23,542	23,499	76	3.45	2.80 ×10^−4^	K4.2	388	K5	2322
25,733..25,809	25,766	77	2.2	0.01	K4.2	2654	K5	55
26,192..26,260	26,224	69	1.49	0.07	K4.2	3113	K5	0
29,571..29,639	29,597	69	1.46	0.07	HHV8_gs01	0	ORF16	602
30,804..30,900	30,852	97	2.56	5.26 × 10^−3^	ORF16	0	ORF17.5	0
43,108..43,187	43,141	80	1.67	0.05	ORF25	0	ORF26	3844
58,909..58,982	58,937	74	3.85	5.96 × 10^−5^	ORF38	0	ORF39	17
59,732..59,873	59,776	142	1.69	0.05	ORF38	757	ORF39	0
62,560..62,679	62,617	120	1.36	0.09	ORF40	0	ORF42	0
68,328..68,408	68,366	81	2.62	4.43 × 10^−3^	ORF44	928	ORF45	0
85,620..85,692	85,652	73	1.69	0.05	vIRF-1	204	vIRF-4	413
90,922..90,992	90,947	71	2.34	9.77 × 10^−3^	vIRF-4	1749	vIRF-3	0
117,500..117,586	117,539	87	1.84	0.03	HHV8GK18_gp78	0	HHV8GK18_gp79	0
117,923..118,023	117,975	101	2.04	0.02	HHV8GK18_gp78	408	HHV8GK18_gp79	0
119,643..119,720	119,675	78	4.04	2.69 × 10^−5^	HHV8_gs02	35	HHV8_gs03	513
124,185..124,263	124,225	79	1.69	0.05	HHV8GK18_gp82	369	HHV8GK18_gp81	0
126,244..126,323	126,287	80	4.94	3.98 × 10^−7^	HHV8GK18_gp82	2428	HHV8GK18_gp81	0
129,824..129,931	129,879	108	2.17	0.02	HHV8GK18_gp84	0	HHV8GK18_gp86	723
135,229..135,343	135,296	115	2.9	1.85 × 10^−3^	HHV8GK18_gp86	639	K15	0
135,467..135,558	135,506	92	2.66	3.92 × 10^−3^	HHV8GK18_gp86	877	K15	0
135,919..135,992	135,968	74	1.35	0.09	HHV8GK18_gp86	1329	K15	0
136,375..136,434	136,403	60	1.36	0.09	K15	0		

## Data Availability

All the relevant data are provided in the main text and supplementary file of the manuscript. The ChIP sequencing raw data and processed files are submitted to GEO which is available under accession number GSE202670.

## Supplementary Materials

The following supporting information can be downloaded at: https://www.mdpi.com/article/10.3390/cancers14215396/s1, Figure S1: Confirmation of hypoxic induction in BC3 cells and validation of the ChIP-seq data for non-specific binding; Figure S2: Confirmation of hypoxic induction in cells growing under 1%O2 condition; Table S1: List of primers used to validate ChIP-Sequencing results by ChIP-qPCR. File S1: Original western blots.
