# Supplementary material for: Epigenetic Reprogramming of Kaposi’s Sarcoma-Associated Herpesvirus during Hypoxic Reactivation
Source: Cancers (Basel). 2022 Nov 2;14(21):5396. doi: 10.3390/cancers14215396 (PMC9654037; doi:10.3390/cancers14215396)

Fig 2A H3K4Me3

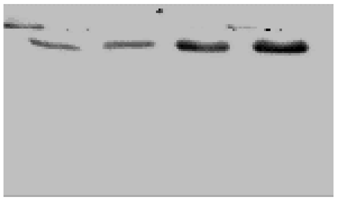

Fig 2A H3K9Me3

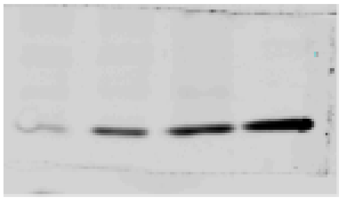

Fig 2A H3K27Me3

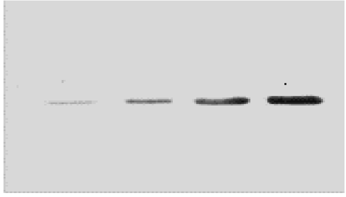

Fig 2A GAPDH

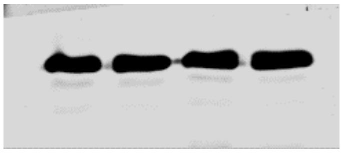

Fig 2B H3K4Me3

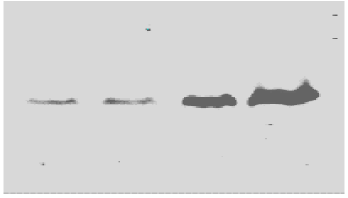

Fig 2B H3K9Me3

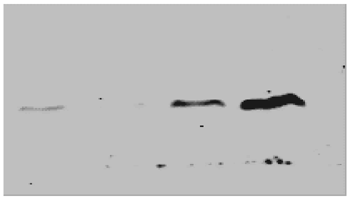

Fig 2B H3K27Me3

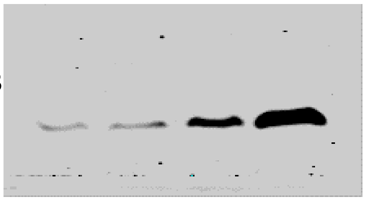

Fig 2B GAPDH

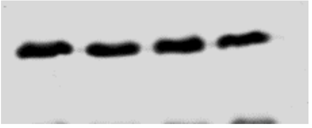

Fig 2C H3K4Me3

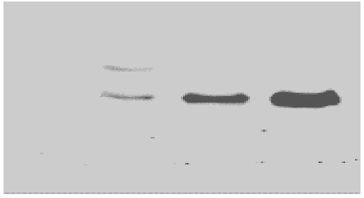

Fig 2C H3K9Me3

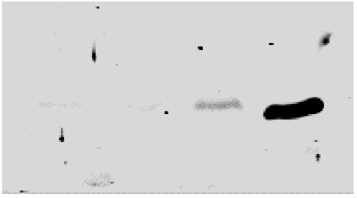

Fig 2C H3K27Me3

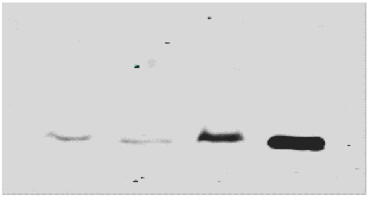

Fig 2C GAPDH

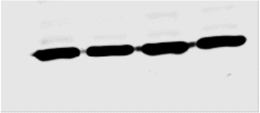

Fig 2D DNMT1

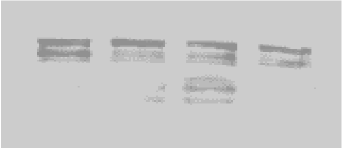

Fig 2D DNMT3A

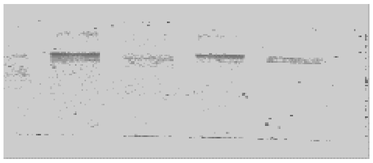

Fig 2D DNMT3B

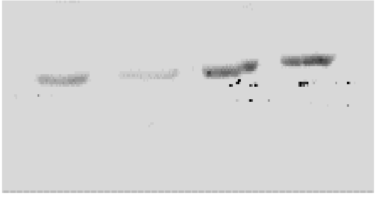

Fig 2D GAPDH

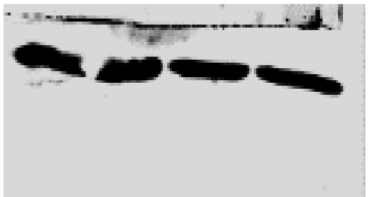

Fig 2E DNMT1

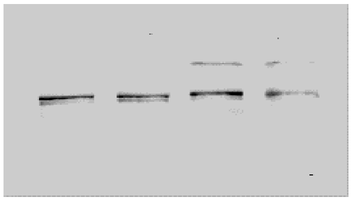

Fig 2E DNMT3A

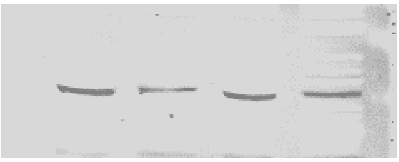

Fig 2E DNMT3B

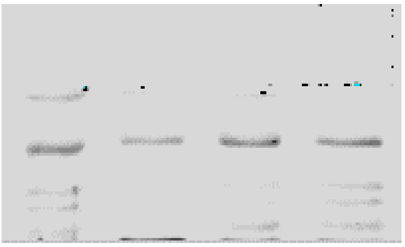

Fig 2E GAPDH

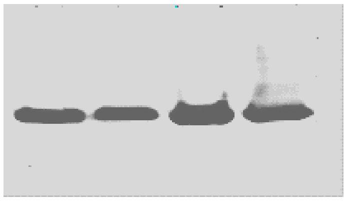

Fig 2F DNMT1

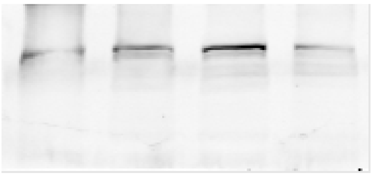

Fig 2F DNMT3A

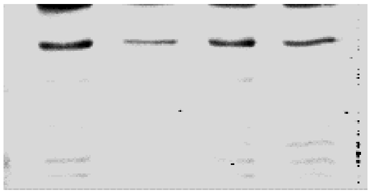

Fig 2F DNMT3B

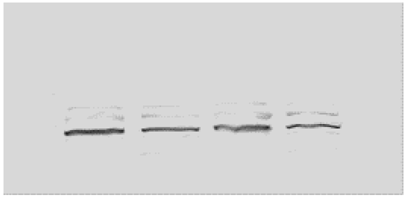

Fig 2F GAPDH

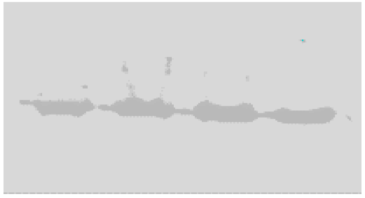

Fig 3A FLAG (LANA)

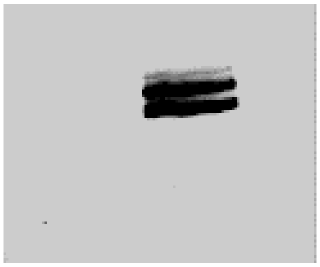

Fig 3A GAPDH for LANA

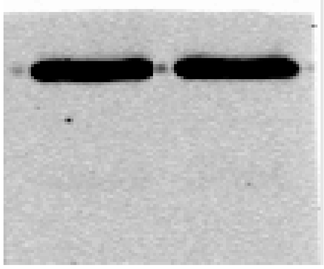

Fig 3A myc for RTA

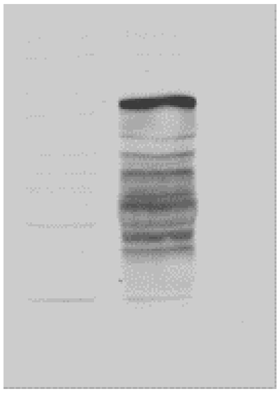

Fig 3A GAPDH for RTA

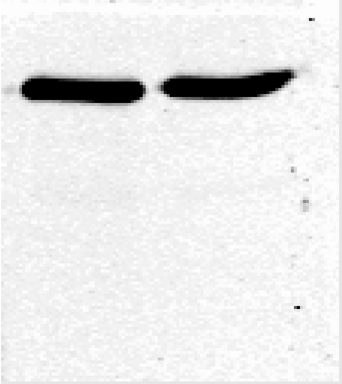

Fig 3A GFP for vCyclin

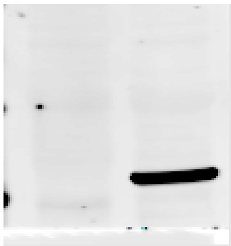

Fig 3A GAPDH for vCyclin

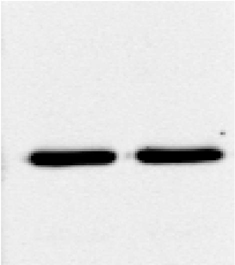

Fig 3A GFP for vFLIP

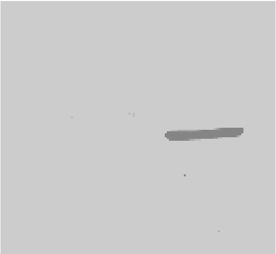

Fig 3A GAPDH for vFLIP

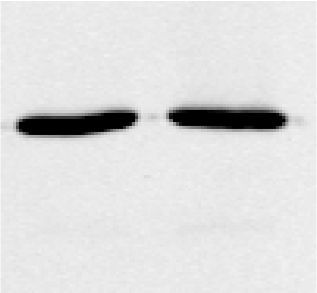

vGPCR

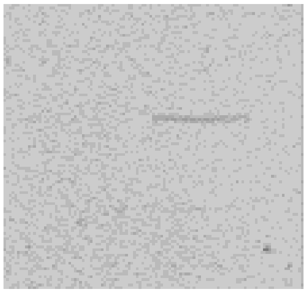

Fig 3A GAPDH for vGPCR

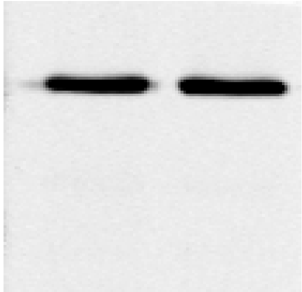

Fig 3B HEK293T H3K4Me3

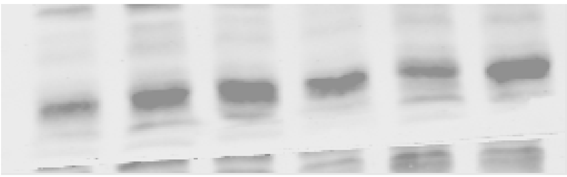

Fig 3B HEK293T H3K9Me3

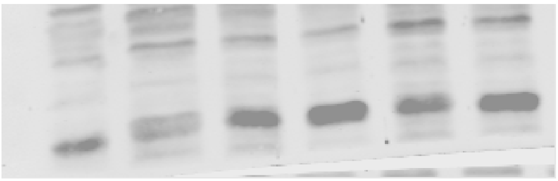

Fig 3B HEK293T H3K27Me3

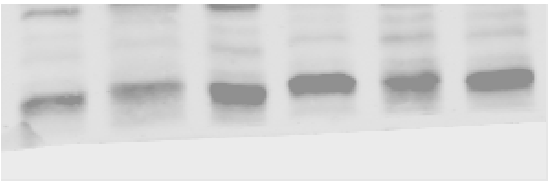

Fig 3B HEK293T DNMT1

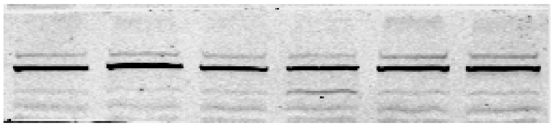

Fig 3B HEK293T DNMT3A

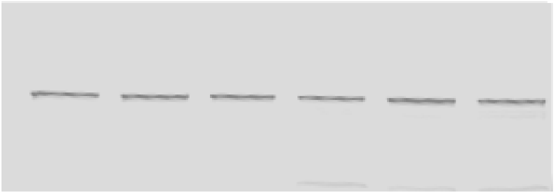

Fig 3B HEK293T DNMT3B

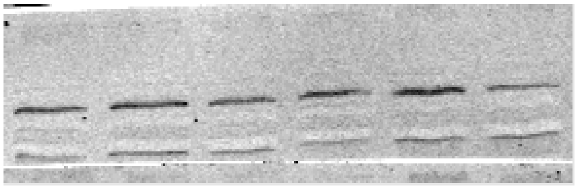

Fig 3B HEK293T GAPDH

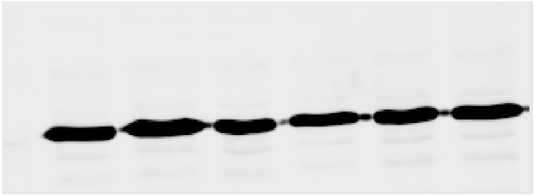

Fig 3B Saos-2 H3K4Me3

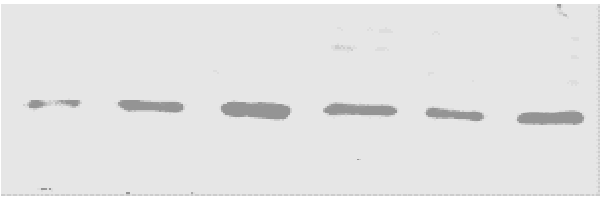

Fig 3B Saos-2 H3K9Me3

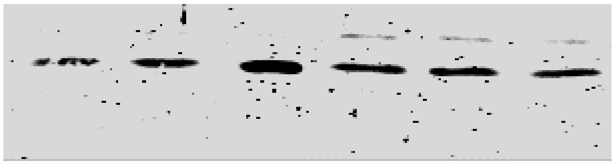

Fig 3B Saos-2 H3K27Me3

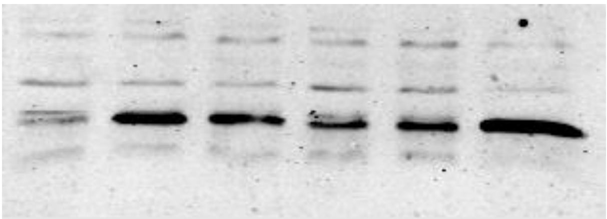

Fig 3B Saos-2 DNMT1

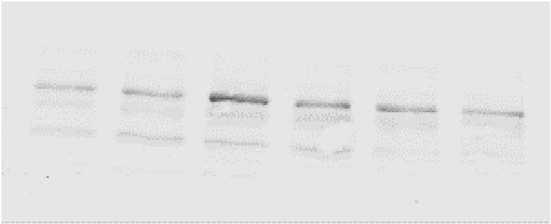

Fig 3B Saos-2 DNMT3A

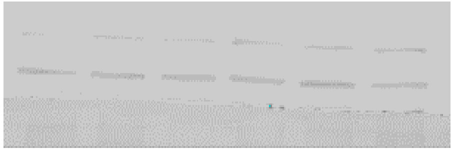

Fig 3B Saos-2 DNMT3B

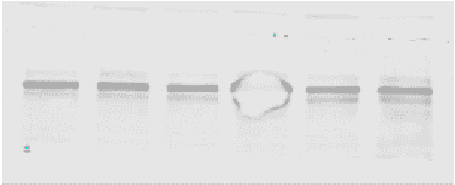

Fig 3B Saos-2 GAPDH

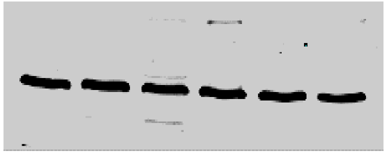

Suppl. Fig. 1A H F1 lp

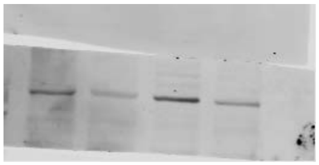

Suppl. Fig. 1A GAPDH

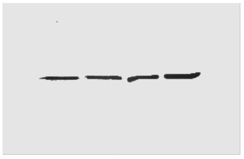

Supplement: Supplementary file 1 [file cancers-14-05396-s001.zip › cancers-1924915-File S1.pdf]
