# Supplementary material for: Epigenetic Reprogramming of Kaposi’s Sarcoma-Associated Herpesvirus during Hypoxic Reactivation
Source: Cancers (Basel). 2022 Nov 2;14(21):5396. doi: 10.3390/cancers14215396 (PMC9654037; doi:10.3390/cancers14215396)
Supplement: Supplementary file 1 [file cancers-14-05396-s001.zip › cancers-1924915-supplementary.pdf]

Confirmation of hypoxic induction in BC3 cells and validation of the ChIP-seq data for non-specific binding

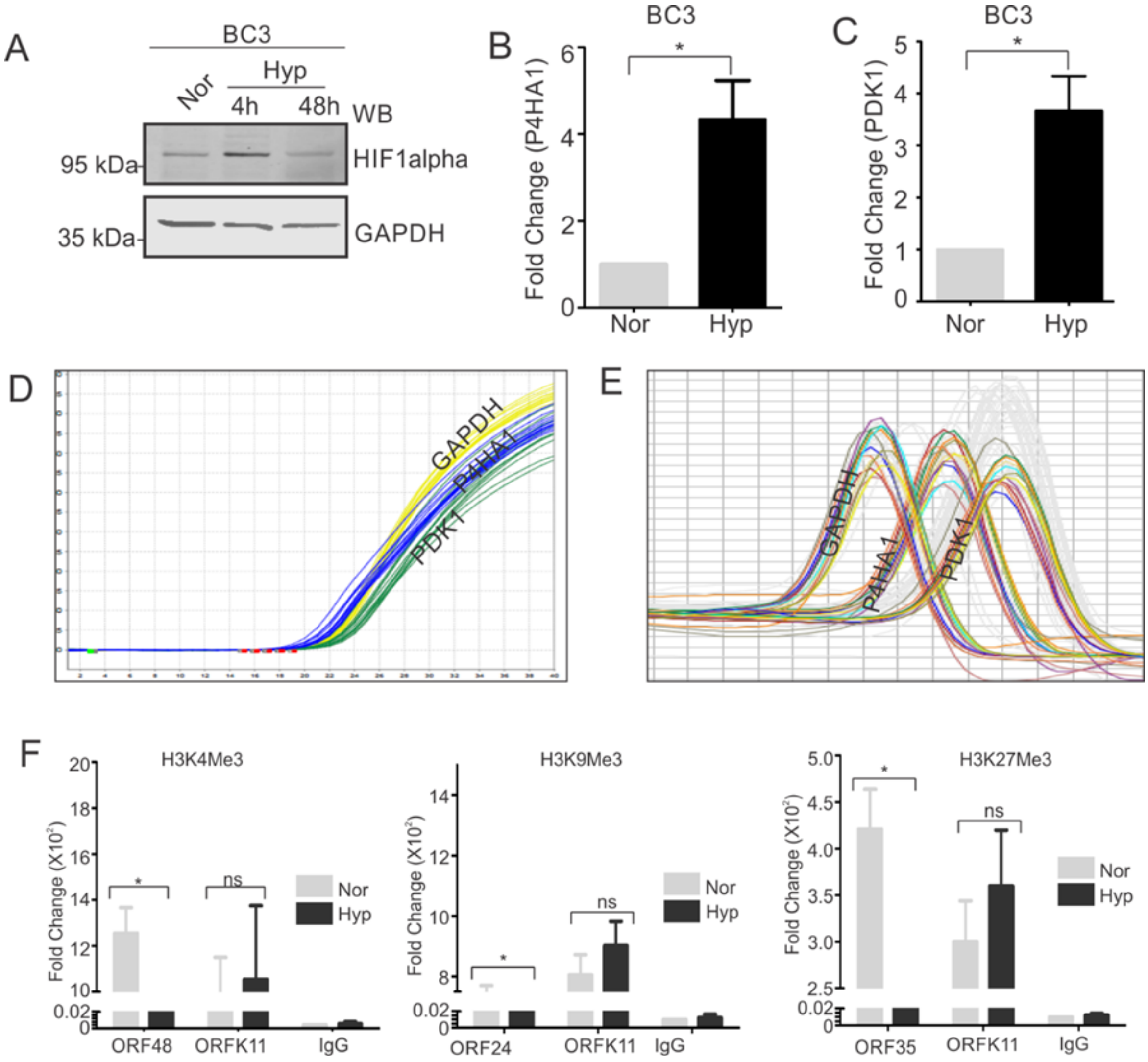

Figure S1. Confirmation of hypoxic induction in cells growing under 1%O_2 condition. (A) HIF1 alpha Western blot analysis in BC3 cell. Cells were grown either in normoxic condition or 1% O2 for 4 and 48 hours. Equal amount of protein was used to blot for HIF1 alpha. GAPDH served as loading control. (B and C) Real-time PCR based confirmation of hypoxia by measuring transcripts of P4HA1 and PDK1 in cells grown under normoxic or hypoxic condition. (D and E) amplification plot and melt curve for the representative set of real-time PCR for P4HA1, PDK1 and GAPDH. (F) ChIP-qPCR for results shown in figure 1A-D in the regions that either remained unchanged or reduced in occupancy. The P value of <0.05 was considered statistically significant. ns, not significant; *, P value < 0.05; **, P value < 0.01;***, P value < 0.005.

Confirmation of hypoxic induction in cells growing under 1%O_2 condition

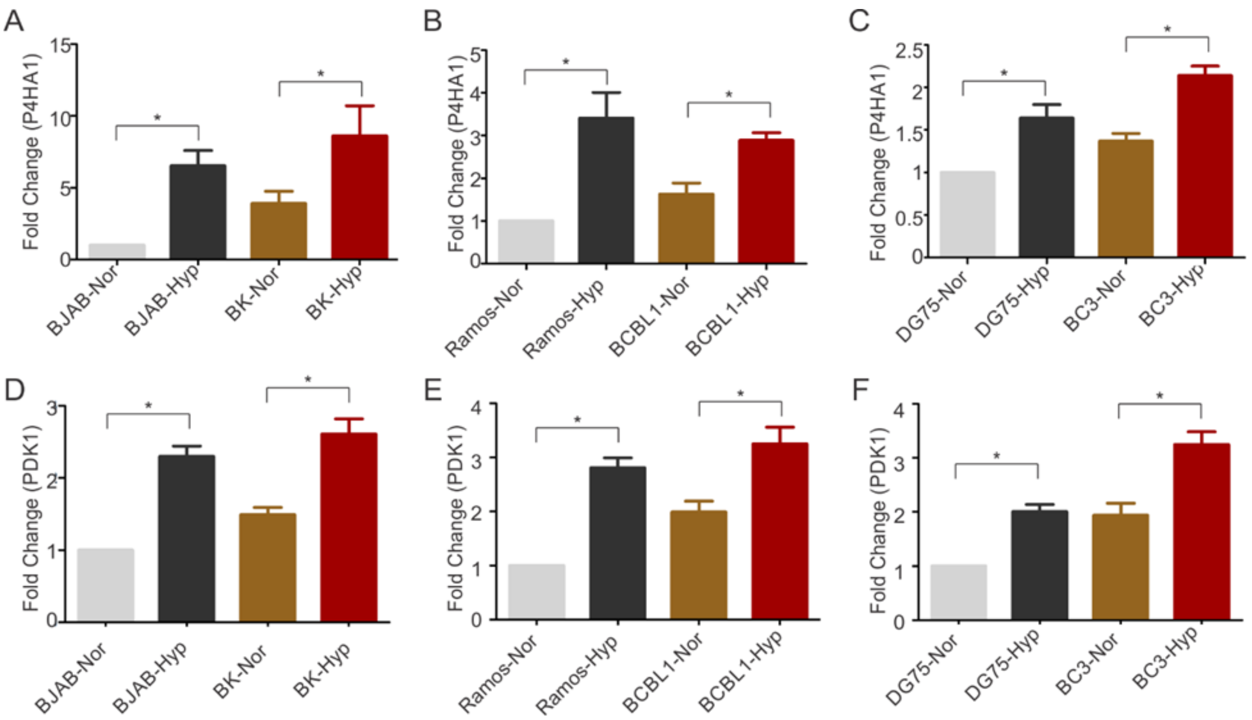

Figure S2. Confirmation of hypoxic induction in cells growing under 1%O_2 condition. (A-F) Real-time PCR based confirmation of hypoxia by measuring transcripts of P4HA1 and PDK1 in BJAB, BJAB-KSHV, Ramos, BCBL1, DG75 and BC3 cells. The P value of <0.05 was considered statistically significant. NS, not significant; *, P value < 0.05; **, P value < 0.01;***, P value < 0.005.

Table S1. List of primers used to validate ChIP-Sequencing results by ChIP-qPCR.

Gene	Forward Primer (5'-3')	Reverse Primer (5'-3')
ORF6	ACCTCTATGCCTATGTGACA	CTTTACATTTAAGGGGAAAT
ORF34	CCTCTTGGTTTCCAACGTGA	CCGCCGCCCCGAAACCCTGC
ORFK12	ATGGATAGAGGCTTAACGGT	CCACGCTGGCCACTCGGGGG
ORF26	GGGGTGCGTATGCTCACGTG	GAAGGTTGTCAGAGGGATCC
ORF29	GCCTATTGGAGTCCCTATCA	GCGGTAGACAGAGCAGGCGT
ORF61	CAGGTCCCTAAGATAGCTCG	TCCTAACAGCCTTTGATTAC
ORFK6	GCGCGGGGTCACTCGTGTCG	CTTGGTCAGCAAAATAACTC
ORF73	CCGCAACACCTTTACCTCCA	TGGAGACTGCGTGGGTGGCA
